# Supplementary material for: rhEGF Treatment Improves EGFR Inhibitor-Induced Skin Barrier and Immune Defects
Source: Cancers (Basel). 2020 Oct 25;12(11):3120. doi: 10.3390/cancers12113120 (PMC7692663; doi:10.3390/cancers12113120)
Supplement: Supplementary file 1 [file cancers-12-03120-s001.pdf]

cancers

MDPI

Supplementary Materials

rhEGF Treatment Improves EGFR Inhibitor-Induced Skin Barrier and Immune Defects

Ji Min Kim, Jun Ho Ji, Young Saing Kim, Suee Lee, Sung Yong Oh, Seok Jae Huh, Choon Hee Son, Jung Hun Kang, So Yun Ahn, Jung Eun Choo, Ki-Hoon Song and Mee Sook Roh

1) β-actin (45 kDa)

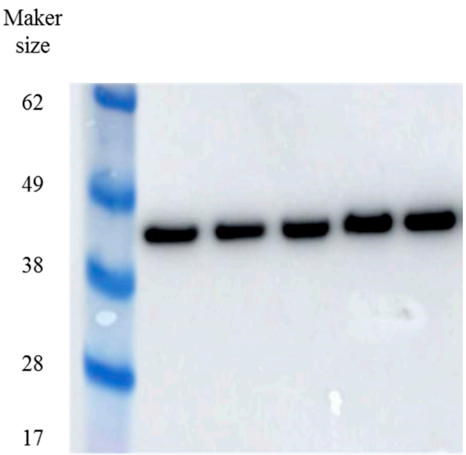

2) EGFR (175 kDa)

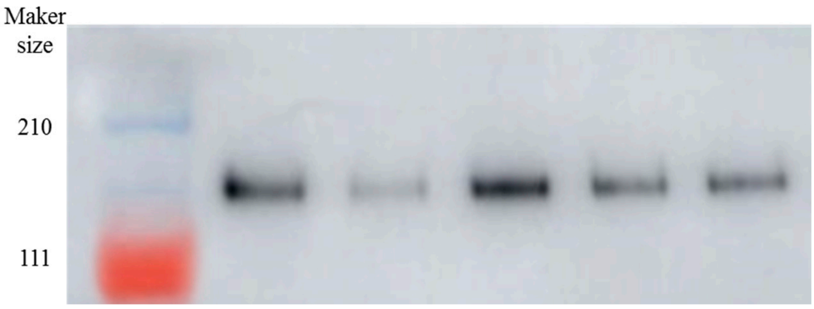

3) pEGFR (175 kDa)

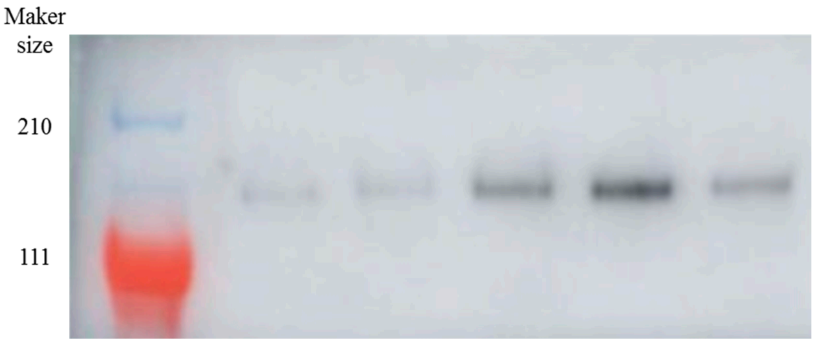

2. Figure 1 (b)

1) β-actin

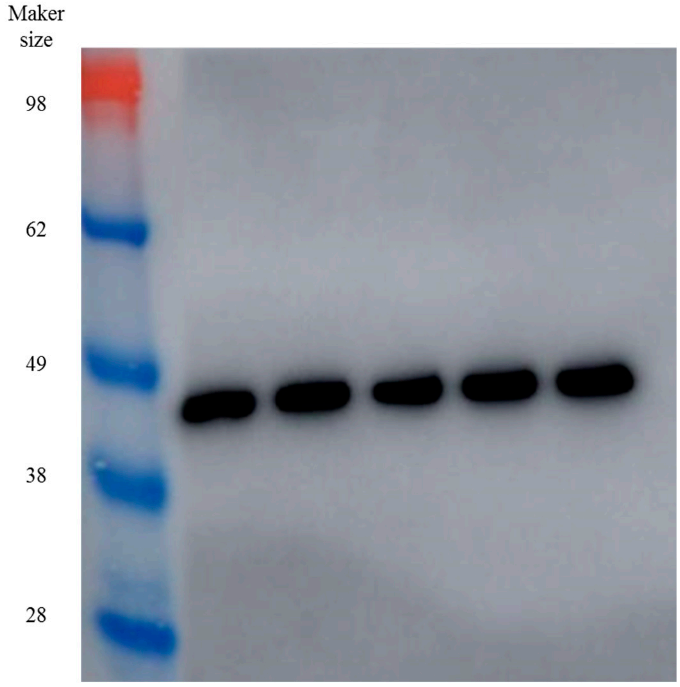

2) AKT (60 kDa)

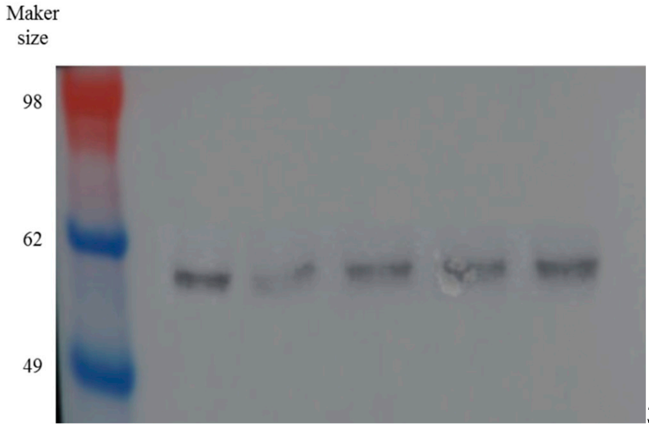

3) pAKT (60 kDa)

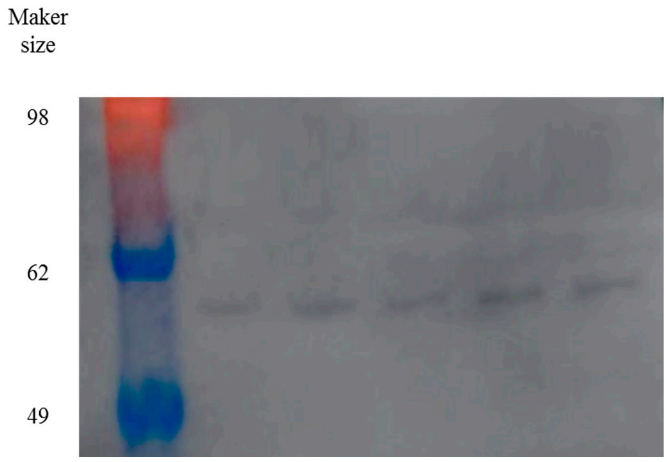

4) PI3K (85 kDa)

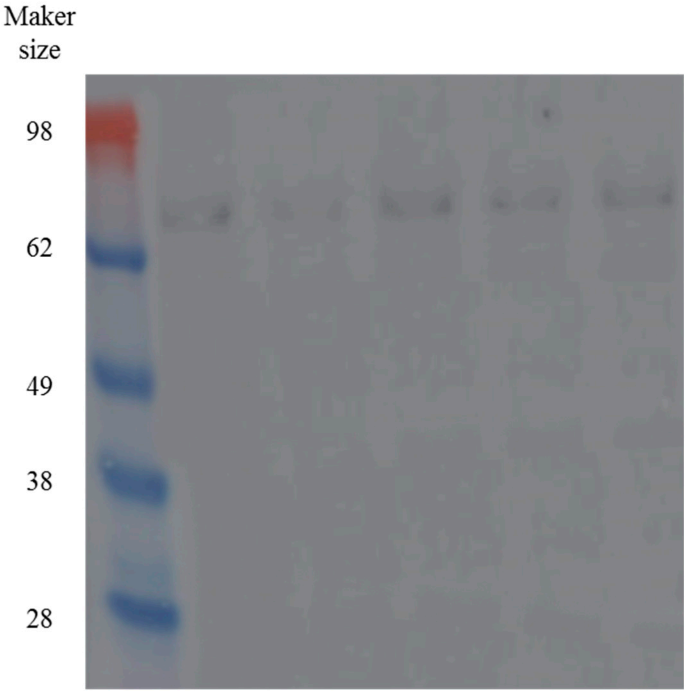

5) pPI3K (60 ~ 85 kDa)

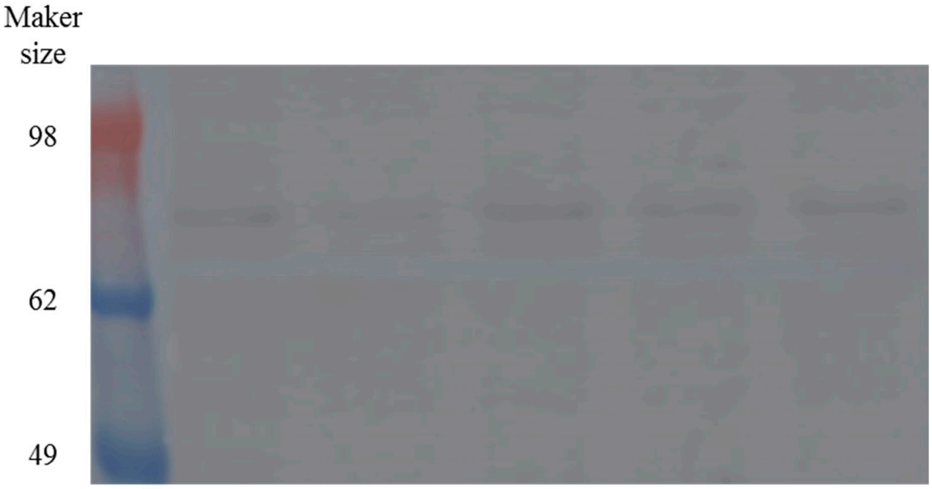

Figure S1: Uncropped Western Blotting figures.

Table S1: List of antibodies

Target protein	Manufacturer	Cat. No.
β-actin	Cell signaling technology	4967S
EGFR		2232S
pEGFR		2235S
AKT		4691S
pAKT		4060S
PI3K		4257S
pPI3K		4228S
Anti-rabbit IgG, HRP-linked Antibody		7074S
